# Anti-Steatotic Effect of *Opuntia stricta* var. *dillenii* Prickly Pear Extracts on Murine and Human Hepatocytes

**DOI:** 10.3390/ijms26072864

**Published:** 2025-03-21

**Authors:** Irene Besné-Eseverri, Jenifer Trepiana, Lina Boutaleb, María Ángeles Martín, Stéphanie Krisa, María Gloria Lobo, M. Pilar Cano, María P. Portillo

**Affiliations:** 1 Nutrition and Obesity Group, Department of Nutrition and Food Sciences, Faculty of Pharmacy, University of the Basque Country (UPV/EHU) and Lucio Lascaray Research Centre, 01006 Vitoria, Spain; irene.besne@ehu.eus (I.B.-E.); mariapuy.portillo@ehu.eus (M.P.P.); 2 CIBER Physiopathology of Obesity and Nutrition (CIBERobn), Institute of Health Carlos III, 28029 Madrid, Spain; 3 BIOARABA Institute of Health, 01006 Vitoria-Gasteiz, Spain; 4 Bordeaux INP, INRAE, Bordeaux Sciences Agro, OENO, UMR 1366, ISVV, University of Bordeaux, F-33140 Villenave d’Ornon, France; lina.boutaleb@u-bordeaux.fr (L.B.); stephanie.krisa@u-bordeaux.fr (S.K.); 5 Science and Food Technology and Nutrition Institute (ICTAN-CSIC), 28040 Madrid, Spain; amartina@ictan.csic.es; 6 CIBER Diabetes and Related Metabolic Diseases (CIBERdem), Institute of Health Carlos III, 28029 Madrid, Spain; 7 Department of Crop Production in Tropical and Subtropical Areas, Instituto Canario de Investigaciones Agrarias (ICIA), 38297 Tenerife, Spain; globo@icia.es; 8 Laboratory of Phytochemistry and Plant Food Functionality, Biotechnology and Food Microbiology Department, Institute of Food Science Research (CIAL) (CSIC-UAM), Nicolás Cabrera 9, 28049 Madrid, Spain; mpilar.cano@csic.es

**Keywords:** prickly pear, *Opuntia stricta* var. *dillenii*, liver steatosis, AML12 hepatocytes, in vitro, HepG2 hepatocytes, agricultural wastes

## Abstract

*Opuntia stricta* var. *dillenii* extracts exhibit anti-oxidative and anti-inflammatory properties, which are of significant interest for the prevention and management of metabolic dysfunction-associated fatty liver disease (MAFLD). The present study is the first to investigate the potential anti-steatotic effect of *Opuntia stricta* var. *dillenii* extracts. The aim is to evaluate the anti-steatotic effect of extracts from various parts of the plant (whole fruit, peel, pulp, and the industrial by-product, bagasse) in an in vitro model using both murine AML12 and human HepG2 hepatocytes. Results have demonstrated that all tested extracts, including those from the whole fruit, peel, pulp, and bagasse, exert an anti-steatotic effect. In murine hepatocytes, the whole fruit extract at 100 μg/mL and the peel extract at 10 μg/mL presented the highest capacity to reduce PA-induced triglyceride accumulation. In fact, the peel was the most potent extract, preventing lipid accumulation at the lowest dose used. In human HepG2 hepatocytes, the peel, pulp, and bagasse extracts at 100 μg/mL demonstrated the greatest triglyceride reduction, suggesting that the human model is less responsive. Regarding the main mechanism of action, the peel and pulp extracts seem to inhibit de novo lipogenesis. Additionally, the downregulation of the fatty acid transporter CD36 appears to contribute to the prevention of triglyceride accumulation in both extracts.

## 1. Introduction

Metabolic dysfunction-associated fatty liver disease (MAFLD) affects approximately 30% of the global adult population and is the leading cause of chronic liver disease worldwide [1]. It is an umbrella term that includes various conditions where more than 5% of hepatocytes present steatosis, along with metabolic risk factors, and excludes excessive alcohol consumption or other ongoing hepatic illnesses [2]. It encompasses a spectrum of liver injury states ranging from steatosis to metabolic steatohepatitis, MAFLD-related cirrhosis, and hepatocellular carcinoma [3].

Currently, there are no clinically approved drugs for MAFLD, and the treatment primarily focuses on lifestyle changes, such as diet and exercise, to promote weight loss [4]. However, many patients with MAFLD frequently struggle to maintain these lifestyle modifications. In this scenario, bioactive compounds naturally present in plants and certain foodstuffs may serve as useful complementary tools for preventing and treating this disease. The impact of natural products has been typically assessed in different signaling pathways, which are altered in MAFLD, including those linked to lipid metabolism, oxidative stress, endoplasmic reticulum stress, and lipotoxicity, demonstrating remarkable therapeutic benefits [2].

*Opuntia* cacti (from the *Cactaceae* family) is a significant source of bioactive compounds like carotenoids, amino acids, vitamins, fibers, betalains (betacyanins and betaxanthins), and phenolic compounds [5,6,7]. Reports have shown that extracts of *Opuntia* species have antioxidant, anti-inflammatory, and anti-hyperglycemic properties that are closely related to MAFLD [8,9]. However, data concerning their ability to reduce the accumulation of hepatic triglycerides are scarce, as *Opuntia ficus-indica* is the most studied species [9]. *Opuntia stricta* var. *dillenii* presents antioxidant and anti-inflammatory effects due to its content of betalains (betacyanins and betaxanthins), as well as its content of polyphenols, among which piscidic acid and certain flavonoids stand out. In this regard, this *Opuntia* species is known for its high betanin content, which gives the fruit a purple color [10]. The monophenol characteristics of betanin and reducing intermediates during the oxidation process might give the molecule an increased potential for H-atom or electron donation, which contributes to the antioxidant property of this *Opuntia* variety, indicating possible positive impacts on metabolic diseases [10]. Consequently, the interest of the present study focuses on the anti-steatotic effect of *Opuntia stricta* var. *dillenii* extracts (whole fruit, peel, pulp, and the industrial by-product, bagasse). The effectiveness of the extracts was tested in an in vitro model of hepatic steatosis with both murine AML12 hepatocytes and human HepG2 hepatocytes. Moreover, research into the mechanisms underlying the noticed effects was conducted.

## 2. Results

### 2.1. Cell Viability in AML12 Hepatocytes Treated with Opuntia stricta var. dillenii Extracts

**Figure 1:**
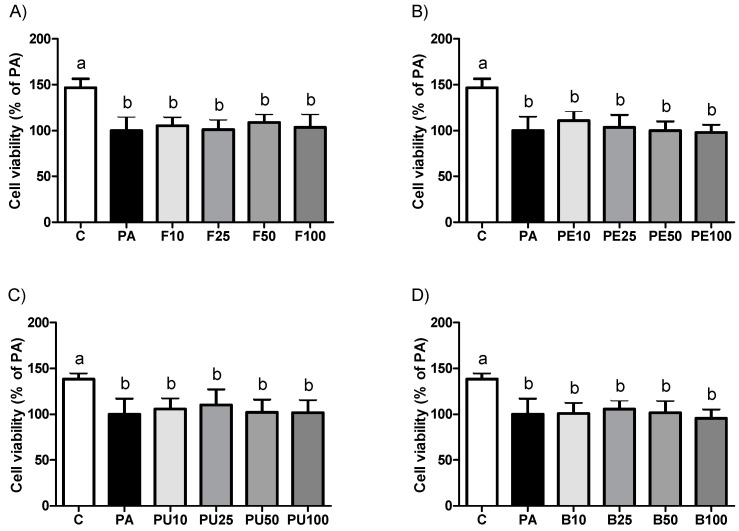
Cell viability in AML12 hepatocytes exposed to 0.3 mM palmitic acid (PA) with or without *Opuntia stricta* var. *dillenii* (**A**) whole fruit (F), (**B**) peel (PE), (**C**) pulp (PU) or (**D**) bagasse (B) extracts at 10 μg/mL, 25 μg/mL, 50 μg/mL or 100 μg/mL for 18 h. Data are means ± SEM. Differences among groups were determined using a one-way ANOVA followed by the Newman—Keuls *post hoc* test. Values not sharing a common letter are significantly different (*p* < 0.05).

First, cell viability was measured in AML12 hepatocytes utilizing *Opuntia stricta* var. *dillenii* extracts to ensure that none of the treatments caused cell damage or toxicity. Results demonstrated that the incubation of hepatocytes with *Opuntia stricta* var. *dillenii* extracts for 18 h did not result in a significant reduction in cell viability across the concentrations tested, compared with the PA group (Figure 1). The incubation of AML12 hepatocytes with PA alone reduced the cell viability by 42.4% relative to the control group.

### 2.2. Triglyceride Levels in AML12 Hepatocytes Treated with Opuntia stricta var. dillenii Extracts

**Figure 2:**
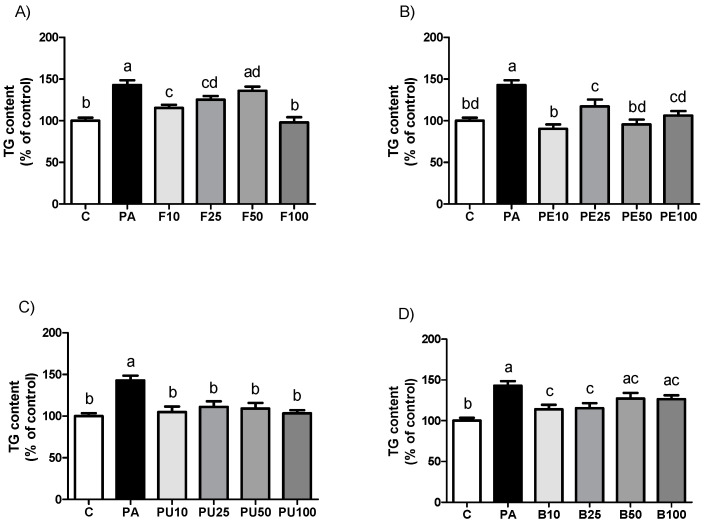
Triglyceride (TG) content in AML12 hepatocytes exposed to 0.3 mM palmitic acid (PA) with or without *Opuntia stricta* var. *dillenii* (**A**) whole fruit (F), (**B**) peel (PE), (**C**) pulp (PU) and (**D**) bagasse (B) extracts, at 10 μg/mL, 25 μg/mL, 50 μg/mL or 100 μg/mL for 18 h. Values are presented as mean ± SEM. Differences among groups were determined using a one-way ANOVA followed by the Newman–Keuls *post hoc* test. Values not sharing a common letter are significantly different (*p* < 0.05).

As represented in Figure 2, all the *Opuntia stricta* var. *dillenii* extracts tested reduced triglyceride accumulation in steatotic AML12 murine hepatocytes (PA-treated cells). When comparing the effectiveness of the four doses used for each derivative, we observed a significant reduction in triglyceride accumulation with the whole fruit extract at 10 µg/mL, 25 µg/mL, and 100 µg/mL. The highest dose caused a significantly greater triglyceride reduction than the other doses, achieving the control value. With the peel extract, the highest effect was induced at 10 µg/mL and 50 µg/mL. All the doses of pulp extract equally prevented triglyceride accumulation since no statistical differences were observed among the tested doses. Lastly, for the bagasse, both 10 µg/mL and 25 µg/mL were equally effective in reducing triglyceride accumulation in the hepatocytes. A tendency towards lower amounts was observed at both 50 µg/mL and 100 µg/mL doses compared to the PA group (*p* = 0.1 and *p* = 0.06, respectively).

Table 1 shows the percentage reduction in triglyceride content versus PA cells for the extracts that caused a significant effect. Statistical analysis was performed to compare treatments with the maximal triglyceride reduction capacity (whole fruit extract at 100 µg/mL, peel extract at 10 µg/mL, pulp extract at 100 µg/mL, and bagasse extract at 10 µg/mL) to identify the most effective treatment. No significant differences were observed between whole fruit at 100 µg/mL and peel extract at 10 µg/mL. The highest effects of the pulp (100 µg/mL) and the bagasse (10 µg/mL) were significantly lower in comparison with the peel extract at 10 µg/mL.

**Figure 3:**
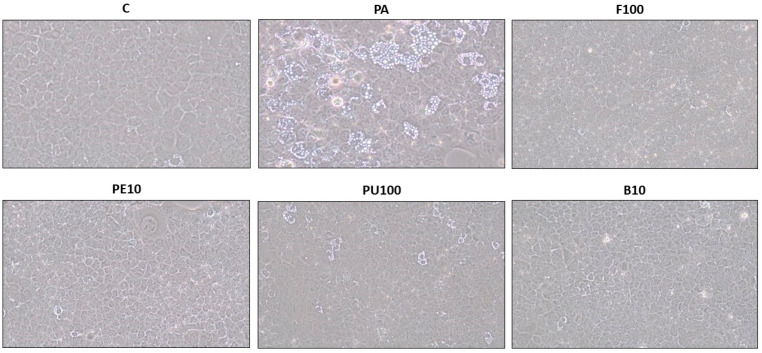
Optical microscopy images (10x objective) illustrating structural features in AML12 hepatocytes exposed to 0.3 mM palmitic acid (PA) with or without *Opuntia stricta* var. *dillenii* whole fruit extract (F, 100 µg/mL), peel extract (PE, 10 µg/mL), pulp extract (PU, 100 µg/mL) and bagasse extract (B, 10 µg/mL) for 18 h.

The optical microscopy analysis revealed that PA treatment induced steatosis in AML12 hepatocytes, whereas co-incubation with the most effective *Opuntia stricta* var. *dillenii* extracts resulted in smaller, more scattered lipid droplets in the cytoplasm (Figure 3).

### 2.3. Cell Viability in HepG2 Hepatocytes Treated with Opuntia stricta var. dillenii Extracts

**Figure 4:**
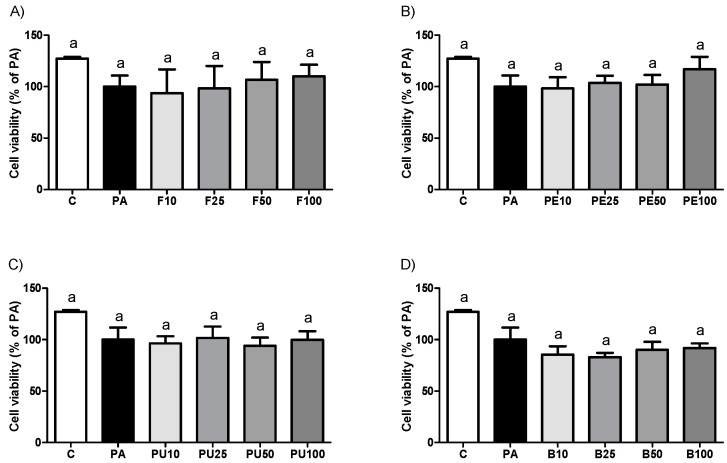
Cell viability in HepG2 hepatocytes exposed to 0.3 mM palmitic acid (PA) with or without *Opuntia stricta* var. *dillenii* (**A**) whole fruit (F), (**B**) peel (PE), (**C**) pulp (PU) or (**D**) bagasse (B) extracts at 10 μg/mL, 25 μg/mL, 50 μg/mL or 100 μg/mL for 18 h. Data are means ± SEM. Differences among groups were determined using a one-way ANOVA followed by the Newman–Keuls *post hoc* test. Values not sharing a common letter are significantly different (*p* < 0.05).

As part of the subsequent phase of our study, we aimed to determine whether the anti-steatotic effects of *Opuntia stricta* var. *dillenii* extracts, observed in murine hepatocytes, could be replicated in human hepatocytes. For this purpose, HepG2 cells, a commonly used cell line in liver research, were employed. Incubation of HepG2 hepatocytes with *Opuntia stricta* var. *dillenii* extracts for 18 h showed no significant decrease in cell viability at any concentrations tested compared to the PA group (Figure 4). It should be noted that exposure to PA alone showed a tendency toward decreased cell viability by 21.5% relative to the C group.

### 2.4. Triglyceride Levels in HepG2 Hepatocytes Treated with Opuntia stricta var. dillenii Extracts

**Figure 5:**
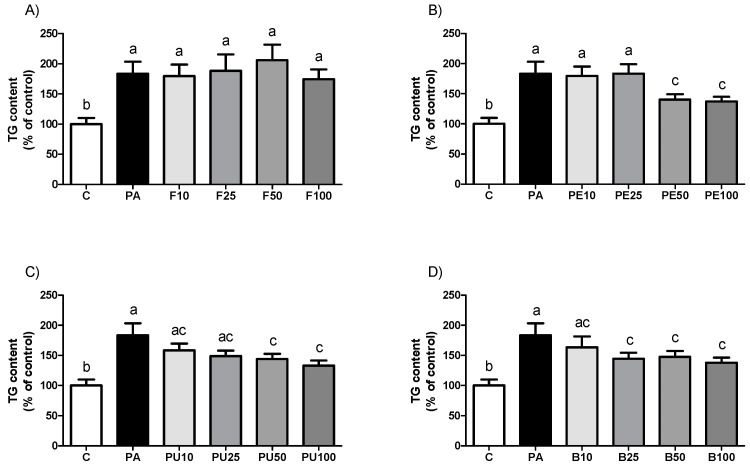
Triglyceride (TG) content in HepG2 hepatocytes exposed to 0.3 mM palmitic acid (PA) with or without *Opuntia stricta* var. *dillenii* (**A**) whole fruit (F), (**B**) peel (PE), (**C**) pulp (PU) and (**D**) bagasse (B) extracts at 10 μg/mL, 25 μg/mL, 50 μg/mL or 100 μg/mL for 18 h. Values are presented as mean ± SEM. Differences among groups were determined using a one-way ANOVA followed by the Newman–Keuls *post hoc* test. Values not sharing a common letter are significantly different (*p* < 0.05).

As shown in Figure 5, when HepG2 hepatocytes were incubated with *Opuntia stricta* var. *dillenii* whole fruit extract, no changes in triglyceride accumulation were observed, regardless of the dose used, compared to the PA cells. Doses of 50 μg/mL and 100 μg/mL of both peel and pulp extracts effectively prevented triglyceride accumulation. Although statistical significance was not reached, a tendency towards lower values was observed with 25 μg/mL pulp treatment (*p* = 0.06). *Opuntia stricta* var. *dillenii* bagasse extract, at doses of 25 μg/mL, 50 μg/mL, and 100 μg/mL, was capable of decreasing triglyceride accumulation in HepG2 cells.

Table 2 displays the percentage reduction in triglyceride content compared to PA cells for the extracts that caused a significant impact. Statistical comparisons were made among the treatments with the maximal triglyceride reduction capacity (peel at 100 µg/mL, pulp at 100 µg/mL, and bagasse at 100 µg/mL) to determine the most effective. No significant differences were noticed between the treatments.

**Figure 6:**
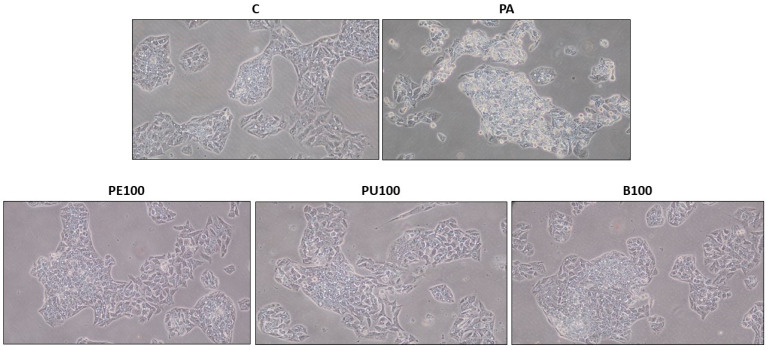
Optical microscopy images (10x objective) showing structural features in HepG2 hepatocytes exposed to 0.3 mM palmitic acid (PA) with or without *Opuntia stricta* var. *dillenii* peel extract (PE, 100 µg/mL), pulp extract (PU, 100 µg/mL) and bagasse extract (B, 100 µg/mL) for 18 h.

The microscopy analysis revealed that treatment of HepG2 hepatocytes with PA-induced steatosis, whereas co-incubation with the most effective *Opuntia stricta* var. *dillenii* extracts resulted in smaller, more dispersed lipid droplets in the cytoplasm (Figure 6).

### 2.5. Effects of Opuntia stricta var. dillenii Extracts on Proteins Involved in Triglyceride Metabolism

**Figure 7:**
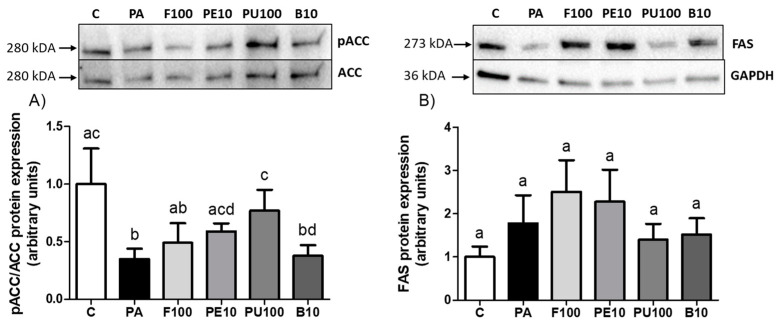
Inactivation rate of ACC (**A**) and protein expression levels of FAS (**B**) in AML12 hepatocytes exposed to 0.3 mM palmitic acid (PA) with or without *Opuntia stricta* var. *dillenii* whole fruit at 100 μg/mL (F100), peel at 10 μg/mL (PE10), pulp at 100 μg/mL (PU100) and bagasse extracts at 10 μg/mL (B10) for 18 h. The Western blot bands shown are representative of six samples/group. Values are presented as mean ± SEM. Differences among groups were determined using a one-way ANOVA followed by the Newman–Keuls *post hoc* test. Values not sharing a common letter are significantly different (*p* < 0.05). ACC: acetyl-CoA carboxylase, FAS: fatty acid synthase, GAPDH: glyceraldehyde 3-phosphate dehydrogenase.

To determine the processes responsible for the lipid-decreasing effect of the tested *Opuntia stricta* var. *dillenii* extracts, the expression of the key proteins implicated in lipid metabolism was measured by Western Blot. Considering that in AML12 hepatocytes, the extracts were able to reduce the TG accumulation to a larger extent and at lower doses compared to HepG2 cells, for this experiment, the AML12 hepatocytes treated with the most effective doses of each extract were selected. Given that ACC is inactivated by phosphorylation, the pACC/ACC ratio serves as an indicator of ACC activity, where a lower ratio reflects enzyme activation. PA cells exhibited significant ACC activation. Both peel and pulp extracts at 10 μg/mL and 100 μg/mL, respectively, partially prevented this effect, as demonstrated by significantly higher pACC/ACC ratio levels in comparison with steatotic cells (Figure 7A, *p* < 0.05). Although the difference in FAS protein expression between C and PA groups did not reach statistical significance (*p* = 0.15), an increase of 78% was observed in PA cells. None of the *Opuntia stricta* var. *dillenii* extracts modified FAS levels compared to PA hepatocytes (Figure 7B).

**Figure 8:**
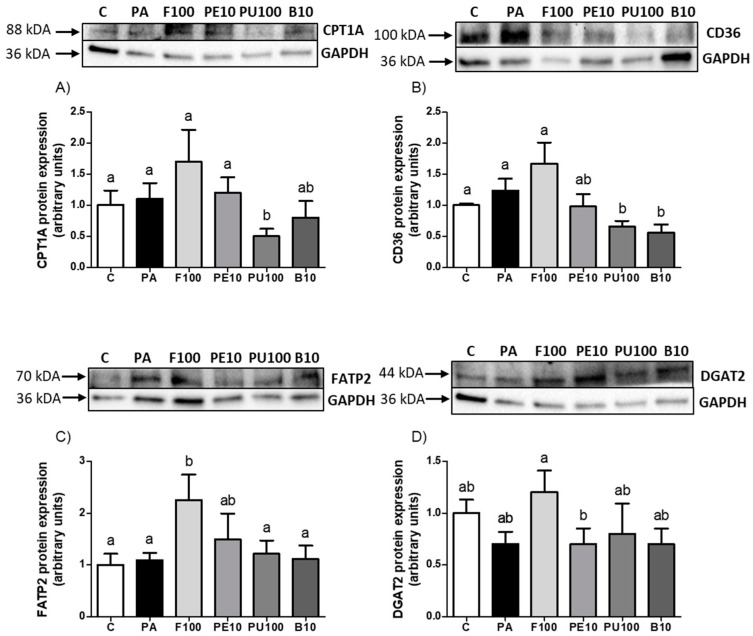
Protein expression levels of CPT1A (**A**), CD36 (**B**), FATP2 (**C**) and DGAT2 (**D**) in AML12 hepatocytes exposed to 0.3 mM palmitic acid (PA) with or without *Opuntia stricta* var. *dillenii* whole fruit at 100 μg/mL (F100), peel at 10 μg/mL (PE10), pulp at 100 μg/mL (PU100) and bagasse extracts at 10 μg/mL (B10) for 18 h. The Western blot bands shown are representative of six samples/group. Values are presented as mean ± SEM. Differences among groups were determined using a one-way ANOVA followed by the Newman–Keuls *post hoc* test. Values not sharing a common letter are significantly different (*p* < 0.05). CD36: CD36 molecule, CPT1A: carnitine palmitoyltransferase 1A, DGAT2: diacylglycerol O-acyltransferase 2, FATP2: solute carrier family 27 member 2, GAPDH: glyceraldehyde 3-phosphate dehydrogenase.

Concerning the lipid oxidation pathway, CPT1A, a key protein for the uptake of long-chain fatty acids into the mitochondrion, was analyzed. PA did not affect CPT1A protein expression, but a significant decrease was observed in *Opuntia stricta* var. *dillenii* pulp extract-treated cells when compared to PA cells (Figure 8A, *p* < 0.05). Although it did not reach statistical significance, it should be noted that fruit extract treatment increased CPT1A levels by 60% compared to PA cells (*p* = 0.14). With the aim of analyzing the uptake of fatty acids through the plasma membrane, the fatty acid transporters CD36 and FATP2 protein expressions were measured. After hepatocyte incubation with PA, no significant differences were noticed in these proteins compared to control cells. Regarding CD36 protein, pulp and bagasse extracts decreased this parameter relative to PA cells (Figure 8B, *p* < 0.05). Regarding FATP2 protein, the whole fruit of *Opuntia stricta* var. *dillenii* showed significantly higher levels than PA cells (Figure 8C, *p* < 0.05). DGAT2 protein, which catalyzes the final step in triglyceride synthesis, was measured in hepatocytes, with no significant differences detected in response to PA treatment (Figure 8D). However, hepatocytes that were administered the whole fruit extract exhibited a tendency towards higher levels compared to PA cells.

## 3. Discussion

The *Opuntia* genus is acknowledged for its antioxidant, anti-inflammatory, and antidiabetic properties, and *Opuntia ficus-indica* is the most studied species [10,11]. Recently, attention has shifted towards investigating other species, such as *Opuntia stricta* var. *dillenii*, which grows wild and is used by food enterprises. Although some studies suggest that this cactus species may exhibit similar health effects to *Opuntia ficus-indica* [12,13], its hepatoprotective capacity has not been extensively studied. To date, only three works have studied the effect of *Opuntia stricta* var. *dillenii* seed oil and hydroalcoholic fruit extracts on chemically induced liver damage (CCl_4_, gentamicin, and acetate) [14,15,16]. In addition, we have recently reported the antioxidant properties of *Opuntia stricta* var. *dillenii* peel extract in a model of diet-induced steatosis, where the extract was able to decrease both the oxidative stress and the apoptotic pathway induced by the diet [17]. Nevertheless, to the best of our knowledge, the present study is the first one investigating the potential anti-steatotic effects of *Opuntia stricta* var. *dillenii* extracts.

*Opuntia* contains substantial quantities of vitamins, carotenoids, fibers, amino acids, betalains, and phenolic compounds, which are suggested to contribute to its health benefits [18]. Interestingly, each *Opuntia* species and each part of the cactus plant shows distinct chemical compositions, potentially leading to different biological effects [18]. In this context, *Opuntia stricta* var. *dillenii* is characterized by being rich in betalains like betanin, neobetanin, and phyllocactin [19]. Notably, neobetanin and phyllocactin are bioactive compounds unique to this *Opuntia* species. Moreover, *Opuntia stricta* var. *dillenii* also contains polyphenols, such as phenolic acids (mainly piscidic acid) and flavonoids, primarily isorhamnetin glycosides [19]. As shown in the Table of Section 4.3, the highest concentrations of polyphenols, piscidic acid, and IG2 were found in the peel extract. In terms of betalains, the levels of betanin and phyllocactin were very similar across the whole fruit, peel, and pulp extracts but significantly lower in the bagasse extract. By contrast, neobetanin was more abundant in the pulp.

When AML12 hepatocytes were incubated with the extracts (whole fruit, pulp, peel, or the industrial by-product, bagasse), all of them induced a significant decrease in triglyceride accumulation in the cells. The doses with the maximal triglyceride reduction capacity for each extract were 100 µg/mL and 10 µg/mL (the lowest dose tested) for the whole fruit and the pulp extracts, respectively, and 10 µg/mL for the peel and bagasse extracts, the whole fruit and peel extracts being the most effective treatments at the mentioned doses. In addition, the peel extract was the most potent, as it caused the strongest effect on triglyceride accumulation prevention at the lowest dose (10 µg/mL) among all the extracts tested. Although the published scientific information regarding the effect of *Opuntia stricta* var. *dillenii* extracts on lipid accumulation is scarce, these results are in line with the reduction in the hepatic lipid content induced by fruits of *Opuntia ficus-indica* extracts on in vivo models of steatosis [20,21]. The result of the present study is noteworthy because the peel is a by-product of the food industry, making its utilization as a source of beneficial bioactive compounds an interesting alternative in the realm of sustainability and the circular economy. On the other hand, the bagasse extract partially prevented triglyceride accumulation in hepatocytes, further highlighting its potential within the circular economy. None of the extracts showed a dose-response pattern. Our research group has also reported this observation using extracts from *Nannochloropsis gaditana* microalgae [22] and bioactive compounds such as resveratrol and its metabolites [23].

From a translational perspective, a second experiment was conducted using the HepG2 hepatocytes, one of the most frequently utilized models in the research of liver disease, to determine whether the anti-steatotic effects of *Opuntia stricta* var. *dillenii* extracts are also found in human cells. In this case, the whole fruit extract did not prevent PA-induced triglyceride accumulation at any of the tested doses. Regarding the peel, pulp, and bagasse extracts, although they partially reduced triglyceride accumulation in hepatocytes, higher doses were generally required to induce a significant effect or achieve the maximal response compared to AML12 hepatocytes. Moreover, the maximal effects reached were lower than those found in AML12 hepatocytes. These results show that the HepG2 cell line is less sensitive to the effects of *Opuntia stricta* var. *dillenii* extracts compared to the murine AML12 line. Other authors have also observed a similar differential response [24,25]. In fact, it has been reported that the lipid and glucose metabolism is quite different between AML12 and HepG2 cell lines, as HepG2 cells are more prone to accumulating lipids, and their beta-oxidation capacity is more restricted. In addition, the AML12 cells are more similar to primary rodent hepatocytes in comparison to the hepatocellular carcinoma-derived HepG2 cells [26]. Thus, these variations between cell lines could be the reason for the differences in the observed effects. Nevertheless, given the limitations of in vitro studies, it is premature to conclude that humans are less responsive than rodents without further in vivo studies in animal models and clinical trials in humans.

In order to describe the mechanisms underlying the decrease in triglyceride accumulation caused by the *Opuntia stricta* var. *dillenii* extracts, their effects on key metabolic pathways involved in fatty acids and triglyceride metabolism were assessed. For this purpose, only the most responsive cell line, AML12 hepatocytes, was used.

Our findings demonstrate that exposing hepatocytes to PA resulted in a significant ACC activation and a tendency toward higher values in FAS protein expression, suggesting an enhancement of the de novo lipogenesis pathway. In an alternative in vitro model of steatosis in AML12 cells, caused by incubation with a similar amount of palmitic acid and glucose, the expression of *Fas*, *Srebp-1c,* and *Scd1* genes was increased, further indicating activation of this metabolic pathway [27,28].

Concerning the mechanism of action triggered by *Opuntia stricta* var. *dillenii* extracts, both the peel at 10 μg/mL and pulp extract at 100 μg/mL were able to increase the pACC/ACC ratio significantly (+67.7% and +118.9%, respectively). Although no changes were observed in FAS protein expression in any group, considering that ACC is the rate-limiting enzyme in the de novo lipogenesis [29], peel and pulp extracts may partially prevent the activation of this metabolic pathway by PA. The down-regulation of de novo lipogenesis implies a decrease in the formation of fatty acids and, thus, in the availability of this lipid species for triglyceride synthesis. The uptake of fatty acids by hepatocytes is mediated by proteins located in the plasma membrane, such as CD36 and FATP2 [30]. In our experiment, we found that co-incubation of pulp at 100 μg/mL and bagasse at 10 μg/mL with PA decreased CD36 protein expression significantly. These findings suggest that for the pulp extract, reduced fatty acid availability for creating triglycerides was due not only to the inhibition of de novo lipogenesis but also to diminished uptake from the bloodstream. Furthermore, in hepatocytes treated with the pulp extract, CPT1A levels were decreased significantly, meaning that a reduction in the β-oxidation pathway may represent a compensatory mechanism rather than a direct mechanism implicated in the reduction in triglyceride accumulation. In the case of the whole fruit, its effectiveness may be supported by a tendency in the up-regulation of CPT1A (+60% increase vs. steatotic hepatocytes), indicating a higher fatty acid influx into the mitochondria for β-oxidation.

Overall, the results from the present study are promising, as *Opuntia stricta* var. *dillenii* extracts have shown a beneficial effect in preventing lipid accumulation in hepatocytes triggered by multiple mechanisms of action. The data regarding the metabolism of the compounds of *Opuntia stricta* var. *dillenii* are scarce. Thus, further research is required to determine the bioavailability of the bioactive compounds in *Opuntia stricta* var. *dillenii* extracts in order to assess their potential for preventing hepatic steatosis. It should be noted that we carried out research to analyze the bioaccessibility of the predominant betalains and phenolic compounds present in *Opuntia stricta* var. *dillenii* using the INFOGEST^®^ methodology. Despite variations by compound and product type, significant losses were observed in both phenolic compounds and betalains [19]. Nevertheless, the findings of the present study provide us with preliminary data for conducting in vivo research using *Opuntia stricta* var. *dillenii* extracts to prevent MAFLD and to analyze changes induced in liver lipid metabolism, oxidative stress, and inflammatory markers in a diet-obesity model.

## 4. Materials and Methods

### 4.1. Reagents

For murine AML12 hepatocytes, Dulbecco’s Modified Eagle’s Minimal Essential Medium (DMEM)/HAM’s F12 (F-12 Nutrient medium) Glutamax was used, while Eagle’s Minimum Essential Medium (MEM) from Corning (New York, NY, USA) was employed for human hepatocellular carcinoma HepG2 cells. For both cell line cultures, fetal bovine serum (FBS) was obtained from Corning, streptomycin-penicillin solution and trypsin/EDTA were purchased from Lonza (Basel, Switzerland), and palmitic acid (PA) from Sigma-Aldrich (St. Louis, MO, USA). For AML12 hepatocytes, insulin, transferrin, and selenium (ITS), acquired from Thermofisher (Waltham, MA, USA), were also used.

### 4.2. Opuntia stricta var. dillenii Extracts

*Opuntia stricta* var. *dillenii* prickly pears were collected in July of 2019 in Tinajo, Lanzarote, The Canary Islands, Spain (29°3′ N, 13°4′ W, 209 m above sea level), and were processed to obtain the peels, pulps, and whole fruit tissues. In addition, the by-product of *O. stricta* var. *dillenii* prickly pear (bagasse), acquired from the jam industry, was supplied by Bernardo’s company (Lanzarote, Spain). Every sample was sliced, frozen using liquid nitrogen (N_2_), freeze-dried, and subsequently pulverized to fine particle size (<2 mm), with the seeds removed. After that, extracts rich in betalains and phenolic compounds were obtained under reduced light conditions from freeze-dried tissue and samples, as previously documented by Gómez-López et al. [19]. In summary, 1 g of each freeze-dried prickly pear tissue was extracted using 5 mL of a 1:1 (*v*/*v*) methanol-water mixture by homogenizing it with a vortex and placing it in an ultrasonic water bath with ice for 4 min. After that, the samples were centrifuged at 10,000 rpm for 10 min at 4 °C, and the supernatants were collected. The extraction process was conducted twice, using 3 mL of a 1:1 (*v*/*v*) methanol-water mixture in each cycle. A final extraction was performed using 3 mL of pure methanol. The solvents in the supernatants were then reduced to a minimal volume using a rotary evaporator (Buchi, Flawil, Switzerland) set at 25 °C. Aliquots of each tissue extract were freeze-dried and kept at −20 °C until needed.

### 4.3. Bioactive Compound Quantification by HPLC

Both betalains and phenolic compounds in *Opuntia stricta* var. *dillenii* fruit extracts were analyzed utilizing HPLC [31]. A 1200 Series Agilent HPLC System (Agilent Technologies, Santa Clara, CA, USA) equipped with a reverse-phase C18 column (Zorbax SB-C18, 250 × 4.6 mm i.d., S-5 µm; Agilent) was used at 25 °C. Betalains and phenolic compounds were identified based on their retention times, UV/Vis, and mass spectral data compared to those of commercial, semi-synthesized, or purified standards. Bioactive compounds were quantified by utilizing calibration curves of their respective isolated and semi-synthesized standards, with five points ranging from 0–300 µg/mL. The amount of each compound was represented in mg/g dry weight (Table 3).

### 4.4. Cell Culture Maintenance

Mouse hepatocytes AML12 (alpha mouse liver 12; ATCC^®^ CRL-2254™) and human hepatocellular carcinoma HepG2 cells (HB-8065™) were obtained from ATCC (Manassas, VA, USA). AML12 cell line was cultured in 75 cm^2^ flasks with DMEM/HAM’s F12 (F-12 Nutrient medium) Glutamax, 10% heat-inactivated FBS, 5 µg/mL insulin, 5 µg/mL transferrin, 5 ng/mL selenium, 40 ng/mL dexamethasone, and 1% penicillin/streptomycin (10,000 U/mL). HepG2 hepatocytes were cultured in 75 cm^2^ flasks with MEM enriched with glucose to match the concentration of DMEM used for AML12 cells, 10% heat-inactivated FBS, and 1% penicillin/streptomycin (10,000 U/mL). The cells were cultured at 37 °C in a humified atmosphere containing 5% CO_2_. When the cell monolayer reached 75% confluence, cells were removed using trypsin-EDTA solution and collected for further experiments.

### 4.5. Experimental Design

Based on previous studies [23], an in vitro model was created to mimic the conditions of hepatocytes in fatty liver was established using mouse AML12 hepatocytes or human hepatocellular carcinoma HepG2 cells, cultured in 6-well plates and treated with 0.3 mM of PA for 18 h to stimulate triglyceride accumulation. The groups treated with the *Opuntia stricta* var. *dillenii* extracts at concentrations of 10, 25, 50, or 100 µg/mL (diluted in distilled H_2_O) were simultaneously coincubated with PA for 18 h. Control cells were treated with an equal volume of the same vehicle. Every experiment was carried out a minimum of three times.

### 4.6. Cell Viability Assay

The crystal violet assay was used to assess live cell count based on cell staining with crystal violet [33]. Briefly, 5 × 10^3^ hepatocytes were plated in 96-well tissue culture plates, and after three days, the cells were exposed to the pertinent compounds for 18 h. Following treatments, cells were washed with phosphate-buffered saline (PBS), fixed in 3.7% formaldehyde, and colored with 0.25% crystal violet for 20 min in the dark. The crystals obtained were then dissolved in 33% acetic acid, and their absorbance was measured at 590 nm using an iMark microplate reader (Bio-Rad, Hercules, CA, USA). Cell viability was indicated as a percentage of control cells.

### 4.7. Optical Microscopy Analysis of Steatotic AML12 and HepG2 Hepatocytes

Lipid droplet accumulation was examined using optical microscopy. AML12 and HepG2 hepatocytes were seeded in 6-well culture plates and exposed to their specific treatments for 18 h before being photographed utilizing an Olympus CH optical microscope (Olympus, Tokyo, Japan), with a 10x objective. ImageJ software 1.53k (NIH, Bethesda, MD, USA) was used to examine the cell features.

### 4.8. Determination of Triglyceride Levels

After each treatment, the medium was removed, and cells were collected for triglyceride measurement. Hepatocytes were thoroughly washed with PBS, then sonicated in a solution containing 10 nM Tris-HCl pH 7.4, 150 nM NaCl, and 1 mM EDTA on ice, using 5-s bursts in a Branson Sonifier SFX550 (St. Louis, MO, USA) fitted with a microtip. Triglyceride levels were assessed using a commercially available kit (Spinreact, Girona, Spain). Protein concentrations were measured using the Bradford method [34]. Triglyceride levels were expressed as mg triglycerides/mg protein and presented as the percentage of the control cells.

### 4.9. Protein Immunodetection

Phospho-acetyl-CoA carboxylase (pACC), total acetyl-CoA carboxylase (ACC), fatty acid synthase (FAS), CD36 molecule (CD36), carnitine palmitoyltransferase 1A (CPT1A), solute carrier family 27 member 2 (FATP2), diacylglycerol O-acyltransferase 2 (DGAT2), and glyceraldehyde 3-phosphate dehydrogenase (GAPDH) were detected by Western Blot analysis.

The samples used for this assay were the doses of each *Opuntia stricta* var. *dillenii* extract that resulted in the greatest reduction in TG content: whole fruit at 100 µg/mL (F100), peel at 10 µg/mL (PE10), pulp at 100 µg/mL (PU100) and bagasse at 10 µg/mL (B10). Cellular protein extracts underwent denaturation at 95 °C for 5 min in Laemmli buffer [35] and then separated through electrophoresis using 4–15% SDS-polyacrylamide gels. The proteins were transferred to polyvinylidene difluoride (PVDF) membranes via electroblotting at a constant amperage (1 mA/cm^2^). Following a 1.5 h block at room temperature with 4% BSA, the membranes were incubated overnight at 4 °C with the following primary antibodies: pACC and ACC (1:1000; Cell Signaling, Danvers, MA, USA), CD36 (1:1000; Cell Signaling, Danvers, MA, USA), CPT1A (1:1000; Abcam, Cambridge, UK), FATP2 (1:1000; Santa Cruz Biotech, Dallas, TX, USA), DGAT2 (1:1000; Abcam, Cambridge, UK) and GAPDH (1:1000; Abcam, Cambridge, UK). After washing, membranes were exposed for 2 h at room temperature to polyclonal anti-mouse (1:5000) (Santa Cruz Biotech, Dallas, TX, USA) for CPT1A and GAPDH, anti-rabbit (1:5000) for pACC, ACC and CD36, and anti-goat (1:5000) for FATP2 and DGAT2. The bound antibodies were visualized with an ECL system (Thermo Fisher Scientific Inc., Rockford, IL, USA), and the blots were scanned using the ChemiDoc™ MP Imaging System (Bio-Rad, Hercules, CA, USA). GAPDH, or the phosphorylated isoforms, served as the loading control.

### 4.10. Statistical Analysis

Data were expressed as mean ± standard error of the mean (SEM) from three independent experiments. Statistical analysis was conducted using SPSS 24.0 (SPSS Inc., Chicago, IL, USA). After confirming the normal distribution of the variables with the Shapiro-Wilks normality test, the effect of treatments in AML12 and HepG2 hepatocytes was assessed using one-way ANOVA followed by Newman–Keuls *post hoc* test, comparing different doses of each extract of *Opuntia stricta* var. *dillenii* with the PA-treated and control cells. The same approach was applied to compare the statistical differences among the treatments that induced maximal triglyceride reduction from each extract. Statistical significance was established at *p* < 0.05.

## 5. Conclusions

In the present research, the anti-steatotic effect of *Opuntia stricta* var. *dillenii* whole fruit, peel, pulp, and bagasse extracts has been demonstrated. In AML12 hepatocytes, among all the extracts tested, the whole fruit at 100 μg/mL and the peel at 10 μg/mL have shown the greatest capacity to reduce PA-induced triglyceride accumulation. In addition, the peel extract was the most potent, as it prevented lipid accumulation at the lowest dose. In human HepG2 hepatocytes, the peel, pulp, and bagasse extracts at 100 μg/mL have demonstrated maximal triglyceride reduction, indicating that the human model is less responsive. Regarding peel and pulp extracts, it is proposed that the mechanism underlying the observed effect is the inhibition of de novo lipogenesis. In the case of the bagasse and pulp extracts, the downregulation of the fatty acid transporter CD36 appears to contribute to the triglyceride-decreasing effect of both extracts. In view of the results, a future in vivo study will be conducted to test the anti-steatotic effect of *Opuntia stricta* var. *dillenii* peel extract and analyze the underlying mechanisms.

## Figures and Tables

**Table 1:** Triglyceride content reduction (expressed as %) relative to PA-treated cells, induced by the effective *Opuntia stricta* var. *dillenii* extracts in AML12 hepatocytes.

Extracts	Triglyceride Content Reduction (%)
Whole fruit (10 µg/mL)	16.9 ± 10.1
Whole fruit (25 µg/mL)	9.4 ± 11.9
Whole fruit (100 µg/mL)	29.5 ± 12.6
Peel (10 µg/mL)	34.7 ± 10.0
Peel (25 µg/mL)	17.8 ± 6.1
Peel (50 µg/mL)	34.3 ± 1.2
Peel (100 µg/mL)	25.4 ± 5.0
Pulp (10 µg/mL)	24.6 ± 11.5
Pulp (25 µg/mL)	21.5 ± 2.4
Pulp (50 µg/mL)	21.9 ± 9.9
Pulp (100 µg/mL)	26.3 ± 5.2
Bagasse (10 µg/mL)	19.8 ± 7.5
Bagasse (25 µg/mL)	18.6 ± 7.4

**Table 2:** Triglyceride content reduction (expressed as %) relative to PA-treated cells, induced by the effective *Opuntia stricta* var. *dillenii* extracts in HepG2 hepatocytes.

Extracts	Triglyceride Content Reduction (%)
Peel (50 µg/mL)	14.5 ± 14.4
Peel (100 µg/mL)	16.3 ± 14.2
Pulp (50 µg/mL)	13.9 ± 13.5
Pulp (100 µg/mL)	21.0 ± 11.6
Bagasse (25 µg/mL)	15.8 ± 9.0
Bagasse (50 µg/mL)	12.8 ± 11.2
Bagasse (100 µg/mL)	17.1 ± 14.0

**Table 3:** Main individual betalain and phenolic compound content (mg/g dry weight) in *Opuntia stricta* var. *dillenii’s* whole fruit, peel, pulp, and by-product (bagasse). Reprinted with permission from Ref. [32].

Compound	Family	*Opuntia stricta* var. *dillenii*			
		Whole Fruit	Peel	Pulp	Bagasse
Piscidic acid	Phenolic acid	1.64 ± 0.09	2.33 ± 0.33	0.62 ± 0.05	1.54 ± 0.05
Betanin	Betalain	2.97 ± 0.01	2.99 ± 0.05	2.91 ± 0.23	0.84 ± 0.02
Isobetanin	Betalain	1.85 ± 0.00	1.65 ± 0.04	2.28 ± 0.19	0.77 ± 0.02
Betanidin	Betalain	0.04 ± 0.00	0.04 ± 0.00	0.04 ± 0.01	0.02 ± 0.00
6′-O-sinapoyl-O-gompherin	Betalain	0.13 ± 0.00	0.14 ± 0.00	0.08 ± 0.00	0.01 ± 0.00
2′-O-apiosyl-4-O-phyllocactin	Betalain	1.29 ± 0.02	1.22 ± 0.02	1.61 ± 0.18	0.58 ± 0.16
5″-O-E-sinapoyl-2′-apyosil-phyllocactin	Betalain	3.14 ± 0.00	3.23 ± 0.13	2.6 ± 0.07	n.d.
Neobetanin	Betalain	1.95 ± 0.02	0.82 ± 0.00	3.26 ± 0.05	1.03 ± 0.07
Quercetin-3-O-rhamnosyl-rutinoside (QG3)	Flavonoid	0.04 ± 0.00	0.07 ± 0.00	n.d.	0.02 ± 0.00
Quercetin glycoside (QG1)-Quercetin hexosyl pentosyl rhamnoside	Flavonoid	0.04 ± 0.00	0.08 ± 0.00	n.d.	0.02 ± 0.00
Quercetin glycoside (QG2)-Quercetin hexose pentoside	Flavonoid	0.02 ± 0.00	0.02 ± 0.00	n.d.	n.d.
Isorhamnetin glucosyl-rhamnosyl-rhamnoside (IG1)	Flavonoid	0.02 ± 0.00	0.03 ± 0.00	n.d.	0.01 ± 0.00
Isorhamnetin glucosyl-rhamnosyl-pentoside (IG2)	Flavonoid	0.29 ± 0.00	0.52 ± 0.02	0.05 ± 0.00	0.18 ± 0.01

Data are expressed as means ± standard deviation (*n* = 3), derived from a minimum of two independent extracts (*n* = 2) with HPLC determinations performed on each occasion (*n* = 2). n.d. not detected.

## Data Availability

The original contributions presented in this study are included in the article/Supplementary Materials. Further inquiries can be directed to the corresponding author(s).

## Supplementary Materials

The supporting information can be downloaded at: www.mdpi.com/article/10.3390/ijms26072864/s1.
