# Supplementary material for: Anti-Steatotic Effect of Opuntia stricta var. dillenii Prickly Pear Extracts on Murine and Human Hepatocytes
Source: Int J Mol Sci. 2025 Mar 21;26(7):2864. doi: 10.3390/ijms26072864 (PMC11988368; doi:10.3390/ijms26072864)
Supplement: Supplementary file 1 [file ijms-26-02864-s001.zip › ijms-3522150-supplementary.docx]

**Supplementary Table 1.** Bioaccessibility of the most abundant betalain and phenolic compounds in *Opuntia stricta* var. *dillenii* fruit tissues (peel, pulp, and whole fruit), jam production products (intermediate juice and jam), and by-product (bagasse) during *in vitro* gastrointestinal static digestion. This table has been published by Gómez-López I., 2021 (doi: [10.3390/foods10071593](https://doi.org/10.3390%2Ffoods10071593)).

| **Bioaccesibility (%)** | | | | | | |
| --- | --- | --- | --- | --- | --- | --- |
| **Compound** | **Fresh Whole Fruit** | **Fresh Peel** | **Fresh Pulp** | **Bagasse By-Product** | **Fresh Pressed Juice** | **Jam** |
| **BETALAINS** |  |  |  |  |  |  |
| Betanin | 22.42 ± 1.29 ^ab^ | 42.58 ± 3.35 ^c^ | 22.95 ± 0.01 ^ab^ | 19.03 ± 1.14 ^a^ | 28.53 ± 2.29 ^b^ | 0 |
| Isobetanin | 22.85 ± 1.72 ^ab^ | 45.67 ± 1.66 ^c^ | 23.6 ± 0.06 ^ab^ | 18.52 ± 1.20 ^a^ | 26.44 ± 2.42 ^b^ | 0 |
| Betanidin | 47.06 ± 0.77 ^d^ | 21.74 ± 1.09 ^b^ | 36.37 ± 0.35 ^c^ | 10.38 ± 0.28 ^a^ | 25.95 ± 3.14 ^b^ | 0 |
| 2´-O-apiosyl-4-O-pyhillocactin | 20.42 ± 4.04 ^c^ | 41.32 ± 0.78 ^d^ | 5.97 ± 0.30 ^b^ | 21.94 ± 0.71 ^a^ | 0 | 0 |
| Neobetanin | 7.63 ± 2.96 ^a^ | 23.26 ± 1.16 ^b^ | 26.22 ± 2.9 ^b^ | 3.08 ± 0.15 ^a^ | 7.20 ± 0.52 ^a^ | 0 |
| **PHENOLIC ACIDS** |  |  |  |  |  |  |
| Piscidic acid | 61.35 ± 3.07 ^e^ | 40.71 ± 2.78 ^d^ | 7.43 ± 0.35 ^b^ | 2.03 ± 0.08 ^a^ | 44.17 ± 1.78 ^d^ | 18.13 ± 0.97 ^c^ |
| **FLAVONOIDS** |  |  |  |  |  |  |
| Quercetin glycoside(QC1)-Quercetin hexosyl pentosyl rhamnoside | 28.33 ± 2.18 ^b^ | 53.72 ± 5.19 ^d^ | 0 | 0 | 45.38 ± 1.78 ^c^ | 7.15 ± 0.36 ^a^ |
| Isorhamnetin glucoxyl-rhamnosyl-rhjamnoside (IG1) | 15.27 ± 0.88 ^b^ | 30.62 ± 0.98 ^c^ | 0 | 0 | 29.43 ± 3.51 ^c^ | 0 |
| Isorhamnetin glucoxyl-  rhamnosyl-pentoside (IG2) | 20.02 ± 2.95 ^b^ | 41.40 ± 1.09 ^c^ | 37.77 ± 1.85 ^c^ | 18.49 ± 1.85 ^b^ | 36.33 ± 1.34 ^c^ | 0.81 ± 0.24 ^a^ |

Results were expressed as mean ± standard deviation (*n* = 4). This came from obtaining at least two independent extracts (*n* = 2) and performing the determinations of each two times (*n* = 2). Superscript small letters indicate statistically significant differences (*p* ≤ 0.05) between tissues, and by-products.
